# Effects of Supplementing Rumen-Protected Methionine and Lysine on Milk Performance and Oxidative Status of Dairy Ewes

**DOI:** 10.3390/antiox10050654

**Published:** 2021-04-23

**Authors:** Alexandros Mavrommatis, Christina Mitsiopoulou, Christos Christodoulou, Paraskevi Kariampa, Marica Simoni, Federico Righi, Eleni Tsiplakou

**Affiliations:** 1 Laboratory of Nutritional Physiology and Feeding, Department of Animal Science, School of Animal Biosciences, Agricultural University of Athens, Iera Odos 75, 11855 Athens, Greece; mavrommatis@aua.gr (A.M.); chr_mitsiopoulou28@hotmail.com (C.M.); c.christodoulou@aua.gr (C.C.); p1552005@aua.gr (P.K.); 2 Department of Veterinary Science, University of Parma, Via del Taglio 10, 43126 Parma, Italy; marica.simoni@unipr.it (M.S.); federico.righi@unipr.it (F.R.)

**Keywords:** amino acids, antioxidants, enzymes, oxidative stress indicators, milk, blood, ewes, malondialdehyde

## Abstract

There is limited information on the impact of dietary supplementation with separate rumen-protected (RP) amino acids (AA), or with their combination, on ewes’ oxidative status. Sixty ewes were divided into five groups; C: basal diet (control); M: basal diet + 6 g/ewe RP methionine; L: basal diet + 5 g/ewe RP lysine; LML: basal diet + 6 g methionine and 5 g lysine/ewe; and HML: basal diet + 12 g methionine + 5 g lysine/ewe. Milk’s fat content increased in RP-AA fed ewes, while that of protein in M and L only. In blood plasma, the malondialdehyde (MDA) content was reduced in the M, LML, and HML compared to C-fed ewes. An increase in glutathione transferase activity in the blood plasma of the M and LML compared to the C and HML-fed ewes were found. In milk, lower values of the ferric reducing ability of plasma (FRAP) in the LML and HML-fed ewes and of 2,2′-Azino-bis 3-ethylbenzthiazoline-6-sulfonic acid (ABTS) in LML only, were found. Lysine increased milk’s FRAP values and MDA content. Both L and HML diets increased milk’s protein carbonyls content. Methionine improves the organism’s oxidative status, without adversely affecting milk’s oxidative stability. Lysine dietary inclusion affects negatively the oxidative stability of milk.

## 1. Introduction

Proteins and amino acids (AA) constitute a historical scientific issue in ruminant nutrition that counts more than 100 years [1]. The AAs have been studied in high-yielding ruminants, as feed additives capable of fulfilling their protein requirements [2,3] and improve nitrogen efficiency resulting in lowering nutritional cost and minimizing the environmental burden [4]. Further to this, AAs such as methionine are involved in several cells’ metabolic pathways including that of energy [5] since are principal generators of one-carbon compounds [6]. For this reason, AAs can improve energy supply [7] and consequently affect milk performance, chemical composition, and fatty acids (FA) profile [3]. Specifically, it has been reported that methionine is indirectly involved in long-chain FA (LCFA) transportation from blood to udder through the very-low-density lipoproteins (VLDL) [8]. Additionally, methionine has a pivotal role in the de novo short- and medium-chain FA synthesis in mammary cells, since it is a methyl donor for the transmethylation reactions in the biosynthesis of lipids [3]. Moreover, methionine and lysine are widely known as the first limiting AAs for milk protein synthesis in dairy ruminants as they constitute building blocks for caseins synthesis [7,9]. In this light, an increase in milk casein content has been related to improved cheese manufacturing assets in milk, including firmer curd formation and a shorter rennet coagulation interval [10].

Moreover, AAs are involved also, in cellular oxidative balance [6] since participate in taurine and glutathione (GSH) synthesis [11]. GSH is involved in both cellular detoxification through Glutathione transferase (GST) action and hydrogen peroxide neutralization via Glutathione peroxidase (GSH-Px), while Glutathione reductase (GR) is capable of regenerating the GSH by reducing its oxidized form (GSSG). Furthermore, methionine supplementation has been proven to increase the VLDL resulting in improved vitamin E circulation [12]. Hence, the dietary supplementation with rumen-protected amino acids (RP-AA) could suppress the detrimental effect of lipid peroxidation by-products such as malondialdehyde (MDA) [12].

Up to now, the dietary inclusion of rumen-protected methionine and lysine has been well documented in dairy cows mainly focusing on milk performance [13,14], immune response [13,15], oxidative status [16], and reproduction [13,17]. Recently, limiting AAs have been tested as immune function regulators during the early lactation of dairy sheep [18,19], while extensive studies have been carried out on small ruminants’ milk performance [20]. However, to the best of our knowledge, scarce information exists about the optimum inclusion level and the synergetic effect of RP-AAs on milk chemical composition, and oxidative status of both organism and milk in ewes.

Considering the aforementioned, this study aimed to investigate the effect of dietary supplementation with RP methionine or lysine, as well as with a combination of these AAs in two different proportions, on (a) milk yield, chemical composition, fatty acid profile, (b) GSH-Px, GR, GST, catalase (CAT), and superoxide dismutase (SOD), activities in blood plasma and SOD, GR and lactoperoxidase (LPO) activities in milk and c) total antioxidant capacity and oxidative stress indicators [MDA and protein carbonyls (PCs)] in both blood plasma and milk of early lactating ewes.

## 2. Materials and Methods

### 2.1. Diets and Experimental Design

This study continued the analytical approach initiated in previous research works [18,19]. The study was conducted with respect to the guidelines of the European Union Directive on the defense of animals used for scientific purposes (EU 63/2010; Council of the European Union 2010). Briefly, sixty, two- to three-year-old dairy ewes of pure Chios breed, at 50 days in milk (DIM) were grouped into 5 homogenous subgroups (n = 12 ewes/treatment) based on their fat corrected milk (FCM_6%_) yields (2.20 ± 0.39 kg/day), ages, and body weights (BW; 63 ± 6 kg). Each group was allocated to one of the following five groups: C: basal diet (control); M: basal diet + 6 g/ewe RP methionine (MetaSmart™); L: basal diet + 5 g/ewe RP lysine (LysiGEM™); LML: basal diet + 6 g/ewe MetaSmart™ + 5 g/ewe LysiGEM™; and HML: basal diet + 12 g/ewe MetaSmart™ + 5 g/ewe LysiGEM™. MetaSmart™ and LysiGEM™ were incorporated into the concentrates. The basal diet consisted of 1.5 kg of concentrate, 1.1 kg of alfalfa hay, and 0.6 kg of wheat straw/ewe and fulfilled the average nutritional requirements of each dietary group. The forages (alfalfa hay and wheat straw) were provided separately from the concentrates. 

The diets were formulated by NDS Professional software (Ver. 3.9.7.11, Rumen Sas, Reggio Emilia, Italy) using the equations for small ruminants [21]. Animals were fed on a group basis, considering their average energy and nutritional requirements, in order the experimental design to represent the typical commercial farm feeding management and the results to have practical implications for small ruminants. Parturitions in small ruminants are in fact gathered in a very short interval which means that the animals have similar requirements and consequently feed intakes. The available feeding space was higher than the one recommended for adult housed ewes (0.33 m per animal) considering in order to favor simultaneous access and lower competitive interactions at the feeder among animals. Forage was provided with the concentrate in three equal portions after milking while no concentrate was offered in the milking parlor. Diet consumption was being recorded on daily basis ensuring no refusals occurred. 

The RP-AA contained 60% isopropyl ester of hydroxyl analog of methionine, and 68% l-Lysine monohydrochloride, respectively. Therefore, the mean daily intake of ewes that were fed the commercial products of RP methionine and lysine were 3.6 and 3.4 g respectively. The diet composition is presented in Table 1, while further justifications about amino acids chosen levels and ratios are described by [18]. The whole experimental period lasted 75 days.

### 2.2. Sample Collection

The ewes were milked three times per day (06:00, 13:00, and 21:00) with a milking machine. Individual milk samples (*n* = 180; 12 ewes/group × 5 groups × 3 sampling times) were collected on the 25th, 50th, and 75th experimental day and used for milk chemical composition, fatty acid profile, and oxidative status analyses. Milk yield was recorded at the same experimental days taking into account the three milked quantities, while each of the aforementioned individual milk samples was performed by the mixture of 5% of the milk volume obtained by three milkings aiming to ensure the highest reliability.

Individual blood samples were also taken on the 25th, 50th, and 75th experimental day from the jugular vein of each ewe (*n* = 180) after milking prior to access on feeds. Approximately, 10 mL of whole blood were immediately transferred to heparin-containing tubes (170 units heparin; BD Vacutainer, Plymouth, UK) and stored in an icebox (Thomas Scientific, Swedesboro, NJ, USA) until their transfer to the Laboratory of Nutritional Physiology and Feeding. Then, the blood samples were centrifuged (SL16R, Thermo Fisher Scientific, Waltham, MA, USA) at 2500 rpm for 15 min at 4 °C to separate plasma from the cells.

Milk samples for chemical composition were analyzed on the collection day, while milk and blood plasma samples were stored at −80 °C, prior to fatty acids and oxidative status analyses.

### 2.3. Feed Analyses

Feed chemical composition is presented in Table 1. The analytical procedures have been previously described by Tsiplakou et al. [19].

### 2.4. Milk Chemical Composition and FA Profile

Chemical composition (fat, protein, lactose, total solids, and total solids no-fat) was analyzed using an IR spectrometer (MilkoScan 133; FOSS, Hillerød, Demark) after proper validation by Kjeldahl [22] and Gerber [23] methods. Casein content was analyzed according to the reference method, ISO 17997-1/IDF 29 [24] using a FOSS Kjeltec™ 8400 Analyzer Unit and a FOSS Digestion System DT220 (FOSS, Hillerød, Denmark). Fat corrected milk (FCM_6%_) and energy corrected milk (ECM) yield were calculated using the following formulas:

Fat corrected milk (FCM) in 6% based on the Equation (1)

FCM_6%_ = (0.40 + 0.15 × F) × M (1)

where F = fat content (%) and M = milk yield in kg [3].

Energy corrected milk (ECM) yield based on the Equation (2) [25].

ECM = milk yield x (0.071 × fat (%) + 0.043 × protein (%) + 0.2224) (2)

FA profile was performed using an Agilent 6890 N gas chromatograph equipped with an HP-88 capillary column (60 m × 0.25 mm i.d. with 0.20 µm film thickness, Agilent). Information about the temperature program and standard used are available by Mavrommatis and Tsiplakou [26]. The groups of FA were defined as follow [24]:

Short-Chain Saturated Fatty Acids (SCFA) = C_6:0_ + C_8:0_ + C_10:0_ + C_11:0_ (3)

Medium-Chain Saturated Fatty Acids (MCFA) = C_12:0_ + C_13:0_ + C_14:0_ + C_15:0_ + C_16:0_ (4)

Long-Chain Saturated Fatty Acids (LCFA) = C_17:0_ + C_18:0_ + C_20:0_ (5)

Mono-Unsaturated Fatty Acids (MUFA) = C_14:1_ + C_15:1_ + C_16:1_ + C_17:1_ + _cis-9_ C_18:1_ + _trans-11_ C_18:1_ + trans C_18:1_ (6)

Poly Unsaturated Fatty Acids (PUFA) = _cis-9, trans-11_ C_18:2_ + C_18:2n-6c_ + C_18:2n-6t_ + C_18:3n-3_ + C_18:3n-6_ + C_20:3n-3_ (7)

Saturated Fatty Acids (SFA) = SCFA + MCFA + LCFA(8)

Unsaturated Fatty Acids (UFA) = PUFA + MUFA(9)

Saturated/Unsaturated (S/U) = (SCFA + MCFA + LCFA)/(PUFA + MUFA) (10)

Atherogenicity index (AI) = (C_12:0_ + 4 × C_14:0_ + C_16:0_)/(PUFA + MUFA) (11)

### 2.5. Antioxidant Enzymes Activities and Oxidative Status Indicators

The assays for antioxidant enzyme activities, oxidative stress indicators, and the total antioxidant capacity were performed using a UV/Vis spectrophotometer (GENESYS 180, Thermo Fisher Scientific, Waltham, MA, USA) as previously described [27]. The GSTs activities were recorded by monitoring the conjunction of GSH to 1-chloro-2,4-dinitrobenzene (CDNT) at 340 nm. CAT activity was performed using a commercial spectrophotometric kit (Catalase Assay Kit; CAT100, Sigma-Aldrich, St. Louis, MO, USA). GSH-Px activity was assayed according to Paglia and Valentine [28]. GR activity was performed by measuring the reduction of oxidized glutathione (GSSG) to reduce glutathione in presence of nicotinamide adenine dinucleotide phosphate (NADPH) at 340 nm. SOD activity was recorded by monitoring the inhibition of cytochrome c oxidation at 550 nm. LPO activity in milk was performed by monitoring the oxidation of 2,2′-Azino-bis (3-ethylbenzthiazoline-6-sulfonic acid) ABTS presence of hydrogen peroxide at 340 nm. MDA was measured according to Nielsen et al. [29] with some modifications described by Mavrommatis et al. [30]. The protein carbonyls (PC) were assayed according to the method of Patsoukis et al. [31]. The ABTS [32,33] and the ferric reducing ability of plasma (FRAP) [34] assays were used to assess the total antioxidant capacity.

### 2.6. Statistics

The dataset was evaluated in SPSS.IBM software (Version 20.0. Armonk, NY: IBM Corp.) and the results are depicted as mean ± standard error of means (SEM). The effect of dietary treatment was assessed by performing a GLM for repeated measures analysis of variance. The dietary treatments (T) (T = C, M, L, LML, and HML) were defined as the fixed factor and the sampling time (S) as the repeated measure, while their interactions (T × S) were also assessed, according to the following model:

Υijkl = μ + Ti + Sj + Ak + (T × S)ij + eijkl

where Υijk is the dependent variable, μ the overall mean, Ti the effect of dietary treatment (i = 5; C, M, L, LML, and HML), Sj the effect of sampling time (j = 3; 25th, 45th, and 75th day), Ak the animal’s random effect, (T × S)ij the interaction between dietary treatments and sampling time, and eijk the residual error. A total of 180 observations (12 ewes × 5 dietary groups × 3 sampling times) were emerged by 60 experimental units (ewes) and used for each investigated parameter (except for body weight; two sampling times = 120 observations). Posthoc analysis was applied when appropriate using Tukey’s multiple range test. For all tests, the significance level was set at *p* = 0.05. Simplifying the visualization of the results, GraphPad Prism 6.0 (2012) (GraphPad, San Diego, CA, USA) depicted interleaved bars. Furthermore, a discriminant analysis was also performed (variables were entered independent together) on fatty acids data to establish those variables capable of distinguishing and classifying samples among the five dietary groups. Wilk’s lambda(λ) criterion was used for selecting discriminant variables. Thirty-five variables (FA and group values) were entered to develop a model to discriminate the one-hundred 80 samples. Moreover, correlations between energy corrected milk yield, blood urea and b-hydroxybutyrate concentrations (data were obtained by our previous study [19]) and oxidative status of both blood plasma and milk were explored using Spearman’s correlation coefficients and are presented as a 3-color heat map graph.

## 3. Results

### 3.1. Animals and Milk Performance

The dietary treatments did not affect the milk yield, fat- (FCM), and energy corrected milk (ECM), while a significant reduction on these parameters throughout the experimental period was observed (*p* < 0.001) (Table 2). Milk fat content was significantly increased (*p* < 0.001) in RP-AAs fed ewes, with the highest increase being reported in the HML group (Table 2). Milk protein content increased (*p* = 0.002) in the M and L compared to the C-fed ewes. The total milk casein content was significantly increased in the L compared to the C-fed ewes (*p* = 0.039). Total solids no-fat of milk increased (*p* = 0.011) in the L and HML compared to the C-fed ewes, while the total solids increased (*p* < 0.001) in the milk of supplemented ewes. Furthermore, significant interactions (treatment × sampling time) were observed in milk’s protein and lactose content (Table 2, Figure S1).

### 3.2. Milk Fatty Acids Profile

**Figure 1:**
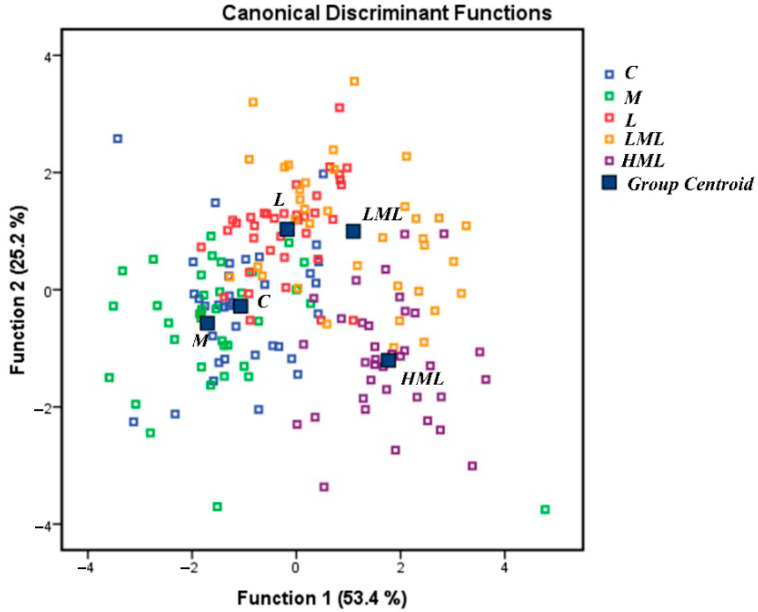
Discriminant plots separating the five dietary treatments (C; blue □, M; green □, L; red □, LML; orange □, and HML; purple □) according to pooled data of three sampling times (25th, 50th, and 75th day) on milk fatty acid profile.

Figure 1 depicts a discriminant plot of the five dietary treatments (C, M, L, LML, and HML) considering the data throughout the experimental period. The proportions of the samples that were correctly classified were 70.6%. Wilks’ (λ) was reported at 0.113 for Function 1 (*p* < 0.001) and 0.307 for Function 2 (*p* < 0.001), while the proportions of C_4:0_, C_14:1_, C_20:0_, C_13:0_, C_6:0_, C_18:3n3_, C_15:0_ and SCFA were the variables that contributed the most. The milk samples of the HML group were located in the lowest right-hand corner of the plot, far away from those of the C, M, and L groups. On the contrary, samples from the LML group were clustered between the aforementioned groups showing a moderate overlapping. Overall, the milk FAs were not considerably altered by RP-AAs supplementation. Confirming this, Table 3 presents minor changes in milk individual FAs. More specifically, the proportions of C_4:0_ and C_6:0_ FA were significantly decreased (*p* < 0.001) in the milk of the HML compared to the C-, M-, L-, and LML-fed ewes, while also significant (*p* < 0.001) interactions between dietary treatment and sampling time were unveiled (Table 3, Figure S2). Hence, the proportion of SCFA was also significantly decreased (*p* = 0.001) in the milk of HML compared to the C-fed ewes. Milk’s C_14:1_ proportion, showed a significant (*p* = 0.013) increase in M-fed ewes, while a decline (*p* = 0.013) was observed in those consuming the LML compared to the C diet. Interestingly, the C_14:1_ proportion decreased (*p* < 0.001) after the 25th experimental day. These fluctuations induced significant (*p* < 0.001) interactions between dietary treatment and sampling time (Table 3, Figure S2). The proportions of C_18:0_ and LCFA tended to increase (*p* = 0.066 and *p* = 0.099 respectively), while that of C_18:1_ *t11* tended to decrease (*p* = 0.068) in the milk of HML compared to the C-fed ewes. The cis-9, trans-11 C_18:2/_trans-11 C_18:1_ Δ^−9^ desaturase index, enhanced significantly in the milk of L in comparison to C-fed ewes (Table 3).

### 3.3. Oxidative Status

**Figure 2:**
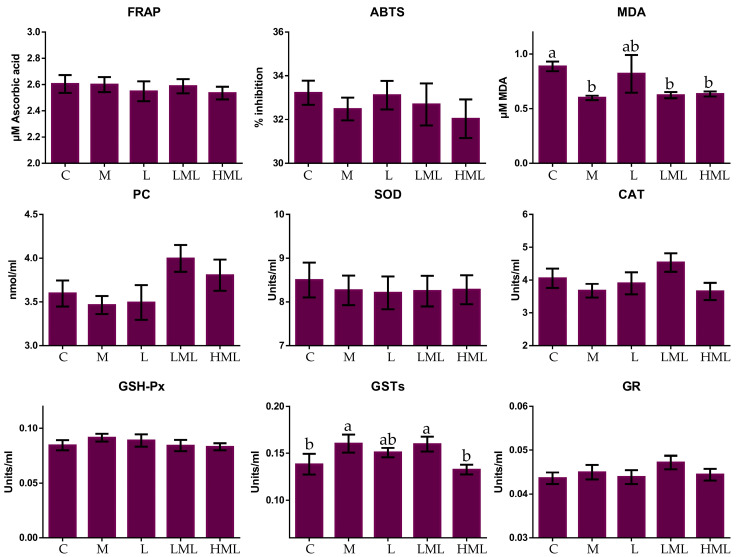
Means ± SEM of total antioxidant capacity, oxidative stress indicators, and enzyme activities (Units/mL), in the blood plasma of ewes, fed the five diets (C, M, L, LML, and HML) within the three sampling times. Bars with different superscript (a, b) between dietary treatments differ significantly (*p* ≤ 0.05).

**Figure 3:**
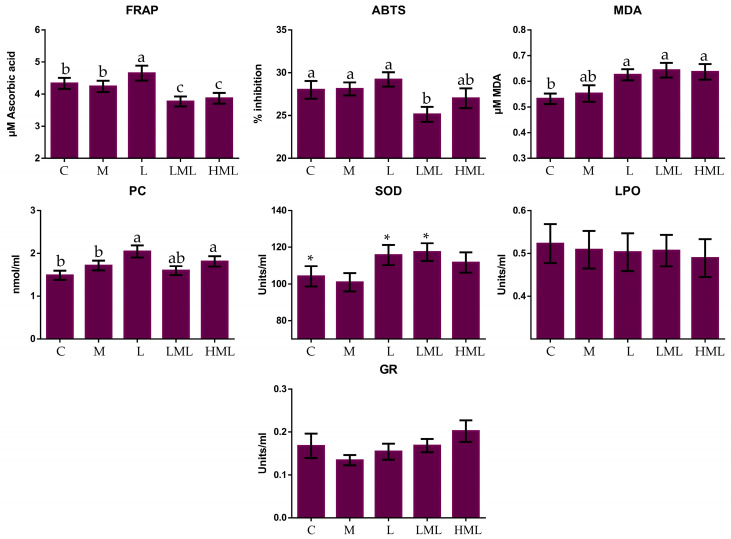
Means ± SEM of total antioxidant capacity, oxidative stress indicators, and enzyme activities (Units/mL), in the milk of ewes, fed the five diets (C, M, L, LML, and HML) within the three sampling times. Bars with different superscript (a, b, c) between dietary treatments differ significantly (*p* ≤ 0.05), while * is referred to *p*-value < 0.10.

Figure 2 and Figure 3 present the total antioxidant capacity, oxidative stress indicators, and antioxidant enzyme activities in blood plasma and milk, respectively. In blood plasma, the malondialdehyde (MDA) content was significantly reduced (*p* = 0.011) in methionine (M, LML, and HML) compared to the un-supplemented fed ewes. A significant increase in GST activity (*p* = 0.007) in the blood plasma of M and LML compared to the C and HML-fed ewes were found. These variations also induced significant (*p* < 0.05) interactions between dietary treatment and sampling time (Table S1, Figure S3). In blood plasma, the highest SOD and GST activities, and total antioxidant capacity, (measured by ABTS assay), were observed on the 75th experimental day (Table S1). The total antioxidant capacity of milk, measured with FRAP assay, was significantly declined in LML and HML compared to the C, M and L-fed ewes (*p* = 0.002), while the opposite was observed when the animals consumed the L diet. Significantly lower, ABTS values in the milk of LML compared to the C, M, and L-fed ewes were found. Lysine inclusion in ewes’ diets caused a significant increase (*p* = 0.048) in milk MDA content. The PCs concentration was elevated (*p* = 0.009) in the milk of L and HML compared to the C- and M-fed ewes. The SOD activity tended (*p* = 0.087) to increase in the milk of L and LML compared to the C-fed ewes. The sampling time affected also, some of the oxidative parameters in ewes’ milk. More specifically, the lowest SOD and LPO activities, and total antioxidant capacity, (measured by FRAP and ABTS assays), were observed at the 75th experimental day while the opposite was found for the PCs content in ewes’ milk (Table S1). These alterations induced significant (*p* < 0.05) interactions between dietary treatment and sampling time for GR and SOD activities, MDA content, and FRAP and ABTS values (Table S1, Figures S3 and S4).

**Figure 4:**
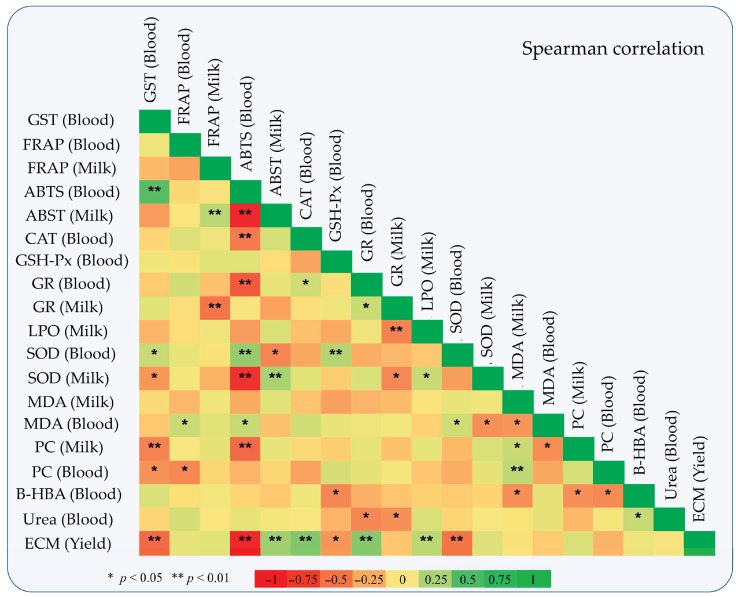
Spearman correlation depicts correlations between oxidative status in blood plasma and milk, b-hydroxybutyrate acid and urea concentration in blood, and ewes’ energy corrected milk yield.

In Figure 4, a Spearman correlation depicts the regression between milk and blood antioxidant indexes, energy, and nitrogen balance metabolites (b-hydroxybutyric acid and urea respectively), and energy corrected milk yield based on the results given by Tsiplakou et al. [19]. Interestingly, ECM yield was negatively correlated with blood GST, GSH-Px, and SOD activities and ABTS values, while a significant positive correlation was observed with milk ABTS values and LPO activity, as well as GR and CAT activities in blood plasma. B-HBA concentration was negatively correlated with GSH-Px activity in blood, MDA levels in milk, and PCs concentration in both milk and blood plasma. Urea in blood was negatively correlated with GR activities in both milk and blood plasma.

## 4. Discussion

### 4.1. Animals’ Performance

In agreement with our findings, the dietary supplementation with RP methionine at 0.2% DM [35] or 2.75 g/animal/day [3] did not affect sheep milk yield. In dairy cows, the dietary supplementation with RP methionine (8, 16, or 24 g/day) increased milk production and its protein content [36]. Additionally, the dietary supplementation with a combination of 18.2 g methionine and 11.7 g lysine daily, increased cow’s milk yield, protein, and caseins content [37], while with RP methionine only (0.17% of DM), enhanced the ECM yield [38]. Discrepancies among studies concerning the effect of dietary supplementation with methionine on milk yield have been reported in a meta-analysis study [39]. On the other hand, it is well documented the improvement of milk protein content by the dietary inclusion of methionine and/or lysine [37], since both of them, are two limiting amino acids for milk protein synthesis [40]. The enhancement of both milk protein and casein content by the RP-AA administration is very important for the dairy industry since affects milk coagulation properties [41].

In this study, the fat content increased in the milk of supplemented ewes. However, the highest fat content was reported in HML-fed ewes indicating that not only the dietary supplementation level of RP-AAs but also the ratio between them determines their effectiveness on milk performance. In agreement with our findings, an increase in milk fat content was observed in RP methionine-fed sheep [42] and goats [43]. On the contrary, some other studies reported no considerable alterations in the milk fat content of goats [44,45] and cows [46,47,48] supplemented with RP methionine. In the mammary gland, AAs can be used as an energy supply as well [49,50]. Indeed, a transcriptomic study by Bionaz et al. [51], indicated the dependence of the tricarbonic acid cycle (TCA) by the AAs as an energy source. Additionally, it was already reported that citrate, an intermediate in the TCA cycle, has a pivotal function in fat synthesis by reducing equivalents in the form of NADPH [52,53].

### 4.2. Fatty Acid Profile

The dietary supplementation with rumen-protected AA had a negligible impact on ewes’ milk FA profile. In accordance with our previous work, the proportion of C_14:1_ increased in M-fed ewes [3]. Additionally, the dietary supplementation with methionine and lysine reduced the proportions of C_4:0_ and C_6:0_ FA, and consequently the short-chain fatty acids, in ewes’ milk, while an opposite trend was observed for the C_18:0_ and LCFA [54]. It is well-justified that low-density lipoprotein, a main source of medium- and LCFA for milk fat synthesis, is strongly dependent on methionine supply through s-adenosyl-methionine (SAM) substrate [54]. Considering the tendency for lower proportions of C_18:1_ *t11* and higher of its biohydrogenation product (C_18:0_) in the higher AA supplementation level (HML) it could be assumed that the increasing levels of nitrogenous compounds within the rumen could upsurge both lipolysis and biohydrogenation function. Remarkably, Giallongo et al. [55] also reported a positive linkage between rumen digestible protein levels and C18-fatty acids biohydrogenation within the rumen. However, further studies are required to validate this hypothesis.

Finally, the highest proportions (but no statistically significant in all groups), of LCFA, C_18:0,_ and *cis-9* C_18:1_ in the milk of AA_s_ fed ewes may be related to the highest fat content which was found in these groups since these fatty acids are able to regulate the fluidity of milk fat globules and consequently enhance milk fat content [26].

### 4.3. Oxidative Status

A significant decline in the MDA content in the blood plasma of methionine-fed ewes (M, LML, HML) was observed accompanied by an increase in GST activity of M- and LML-fed animals. In accordance with our findings, a significant enhancement of GST activity in the intestine and hepatopancreas of methionine-fed juvenile *Jian carp* was found accompanied by a significant reduction in the MDA content of the aforementioned tissues [56]. Moreover, the dietary supplementation with methionine increases GST activity and glutathione concentration in ewes blood plasma [3] and cows’ liver [57], respectively. A significant increase of GST activity in rats’ hepatocytes was also reported when were incubated with l-methionine and l-cysteine simultaneously in vitro [58]. Methionine forms SAM, a precursor for cysteine production [59], which is the limiting AA in the de novo synthesis of GSH [60]. High GSH concentrations protect against cellular damage and tissue degeneration [61]. However, it should be mentioned that the highest inclusion level of methionine in combination with lysine (HML) did not modify the GST activity in ewes’ blood plasma. Similarly, the lowest GSH content in turkeys’ liver was found when their diets were supplemented with the highest methionine content (higher than that recommended by the National Research Council; NRC) [62]. Thus, special attention should be given to the supplemented quantities and in the synergistic effects (ratio) of AAs in animal diets not only to address their optimal inclusion levels but also to avoid physiological imbalance and consequently adverse effects (oxidative stress).

Milk appears to be an optimal environment for MDA production due to its high-fat content. This was reported in cows [63] and lysine-fed ewes (L, LML, HML) of our study. In vitro studies reported that although MDA can react with a broad variety of AA, the epsilon amino group of lysine is the main target [64,65]. Additionally, proteins compared to the free amino acids are more easily damaged by MDA under physiological conditions, as has been indicated in vitro [64,66]. The significant increase in milk PC content of L and HML-fed ewes supports this physiological mechanism. Besides, PCs are formed not only from oxidative deamination of AAs side chains by a direct attack of oxidants but also from products of lipids oxidation [67,68] as indicated by the positive correlation between them which was found in this study. The numerical increase in milk PCs in the M and LML groups may be attributed to methionine inclusion. Without narrowing out the involvement of other AA, methionine can also trigger to some extent the MDA concentration [64,66]. It could be assumed that not only the type of the AA but their mode of action (ratio), as already mentioned in blood plasma, seems to affect differently this metabolic pathway as well. Indeed, the dietary supplementation with these two AAs simultaneously caused the most intense decline in the total antioxidant capacity (FRAP and ABTS) of milk. This indicates that the dietary supplementation with high AA levels, which are involved in cells’ detoxification mechanisms, might have a negative impact on the antioxidant status of milk as previously discussed for the HML-fed ewes’ organism (blood).

## 5. Conclusions

Supplementing ewes’ diet with methionine (6 g/animal/day) not only improved the organism’s oxidative status, but also milk’s chemical composition without affecting the oxidative stability of the latter. Lysine dietary inclusion (5 g/animal/day) induced oxidative stress in ewes’ milk being more intense when combined with methionine at different ratios. More research is needed to define the optimum inclusion levels and the combination (ratio) of RP-AA in ewes’ diets, in order to achieve a positive, synchronize response of the antioxidant defense system of both organism and milk.

## Figures and Tables

**Table 1:** Ration components (% of dry matter; DM) and chemical composition (% of DM) of the feeds administered to the groups (C, M, L, LML, and HML) of ewes involved in the trial.

	Treatment				
	C	M	L	LML	HML
Diet components (% Dry Matter)					
Alfalfa hay 1st cut	35.36	35.3	35.31	35.24	35.17
Wheat straw	19,00	18.96	18.97	18.93	18.89
Concentrate mix	45.63	45.54	45.55	45.46	45.37
Rumen-protected methionine commercial product	-	0.2	-	0.2	0.4
Rumen-protected lysine commercial product	-	-	0.17	0.17	0.17
Diet chemical composition (% Dry Matter)					
Dry Matter	90.39	90.40	90.40	90.41	90.41
Ash	6.85	6.91	6.86	6.92	6.99
Ash-free NDF treated with amylase	38.01	37.93	37.94	37.86	37.86
Acid Detergent Fiber	26.29	26.24	26.24	26.20	26.14
ADL (lignin) *	5.61	5.60	5.60	5.59	5.59
Non Fibrous Carbohydrate	39.69	39.64	39.63	39.57	39.57
Starch *	23.03	23.01	23.00	22.98	22.98
Sugars *	3.22	3.22	3.22	3.21	3.21
Ether Extract	1.67	1.66	1.75	1.75	1.74
Crude Protein	13.92	13.99	13.97	14.03	14.03
Soluble Crude Protein *	3.69	3.78	3.69	3.78	3.78
Rumen Degradable Protein at 3 times the maintenance intake *	8.92	8.90	8.94	8.92	8.92
Methionine %, (g/d) *	0.23 (6.5)	0.35 (7.8)	0.23 (6.5)	0.35 (7.8)	0.35 (9.2)
Methionine, % Metabolizable Protein *	2.18	2.60	2.17	2.59	3.04
Lysine %, (g/d) *	0.60 (20.3)	0.60 (20.3)	0.72 (21.3)	0.72 (21.3)	0.72 (21.3)
Lysine, % Metabolizable Protein *	6.81	6.77	7.12	7.08	7.05
Lysine: Methionine *	3.13:1	2.60:1	3.28:1	2.72:1	2.33:1
Net Energy of lactation (Mcal/kg) *	1.53	1.53	1.53	1.53	1.53

C: basal diet (control); M: basal diet + 6.0 g/ewe RP methionine (MetaSmart™); L: basal diet + 5.0 g/ewe RP lysine (LysiGEM™); LML: basal diet + 6.0 g/ewe MetaSmart™ + 5.0 g/ewe LysiGEM™; and HML: basal diet + 12.0 g/ewe MetaSmart™ + 5.0 g/ewe LysiGEM™. * Estimated using the software NDS Professional Ver. 3.9.7.11, Rumen Sas, Reggio Emilia, Italy.

**Table 2:** Milk performance, chemical composition, and milk’ constituents yield in ewes fed rumen-protected methionine and lysine supplements.

	Treatment (T)						Sampling Time (S)				Effect ^b^		
	C	M	L	LML	HML	SEM ^a^	25	50	75	SEM ^a^	T	S	T × S
Milk yield (kg)	1.68	1.84	1.64	1.72	1.60	0.090	2.13 ^A^	1.52 ^B^	1.44 ^C^	0.058	0.439	<0.001	0.996
FCM_6%_ (kg)	1.60	1.71	1.73	1.82	1.78	0.092	2.17 ^A^	1.62 ^B^	1.42 ^C^	0.045	0.588	<0.001	0.485
ΕCM (kg)	1.46	1.56	1.56	1.64	1.63	0.086	1.92 ^A^	1.49 ^A^	1.30 ^B^	0.040	0.570	<0.001	0.955
Fat content %	5.64 ^c^	6.18 ^b^	6.60 ^ab^	6.34 ^ab^	6.72 ^a^	0.160	6.17 ^B^	6.70 ^A^	6.02 ^B^	0.105	<0.001	<0.001	0.389
Fat yield (g)	94.34	108.18	106.17	110.35	108.58	5.426	129.58 ^A^	101.14 ^B^	85.87 ^C^	3.014	0.216	<0.001	0.223
Protein content %	5.98 ^b^	6.72 ^a^	6.56 ^a^	6.18 ^b^	6.23 ^b^	0.099	6.04 ^B^	6.29 ^A^	6.32 ^A^	0.067	0.002	<0.001	0.001
Protein yield (g)	99.35	112.36	105.40	108.32	100.71	5.028	128.09 ^A^	95.94 ^B^	91.66 ^C^	2.384	0.401	<0.001	0.136
Caseins (%)	4.69 ^b^	4.82 ^ab^	5.16 ^a^	4.89 ^ab^	5.09 ^ab^	0.033	4.83 ^B^	4.98 ^A^	4.98 ^A^	0.017	0.039	<0.001	0.894
Lactose %	5.85	5.87	5.76	5.86	5.83	0.059	6.05 ^A^	5.75 ^B^	5.69 ^C^	0.033	0.661	<0.001	0.003
Total solid %	18.27 ^c^	19.05 ^b^	19.68 ^ab^	19.18 ^b^	19.85 ^a^	0.235	19.10 ^A^	19.68 ^B^	18.85 ^A^	0.133	<0.001	<0.001	0.546
Total solid no-fat %	12.62 ^b^	12.87 ^ab^	13.07 ^a^	12.83 ^ab^	13.12 ^a^	0.104	12.93 ^AB^	12.98 ^A^	12.82 ^B^	0.056	0.011	<0.001	0.606
Body weight (Kg)	63.75	61.79	61.04	61.81	66.33	1.744	62.91	-	62.98	0.792	0.212	0.798	0.510

Means with different superscript letters (a, b, c) between dietary groups and (A, B, C) between sampling time points differ significantly. ^a^ SEM: Standard error of the means. ^b^ Effect: The dietary treatment (T), time (S), and the interaction between dietary treatment × time (T × S) effects were analyzed by ANOVA using a general linear model (GLM) for repeated measures and Post hoc analysis was performed when appropriate using Tukey multiple range test. C: basal diet (control); M: basal diet + 6 g/ewe RP methionine (MetaSmart™); L: basal diet + 5 g/ewe RP lysine (LysiGEM™); LML: basal diet + 6 g/ewe MetaSmart™ + 5 g/ewe LysiGEM™; and HML: basal diet + 12 g/ewe MetaSmart™ + 5 g/ewe LysiGEM™.

**Table 3:** The mean individual fatty acids (FA) (% of total FA), FA groups, S/U ratio, the AI index, and Δ^−9^ desaturase index of ewes’ milk.

	Treatment (T)						Sampling Time ^a^ (S)				Effect ^c^		
	C	M	L	LML	HML	SEM ^b^	25	50	75	SEM ^b^	T	S	T × S
C_4:0_	2.88 ^bc^	3.16 ^a^	3.05 ^ab^	2.67 ^c^	2.05 ^d^	0.077	2.60 ^C^	2.76 ^B^	2.97 ^A^	0.052	<0.001	<0.001	<0.001
C_6:0_	2.77 ^a^	2.80 ^a^	2.81 ^a^	2.68 ^a^	2.28 ^b^	0.056	2.64	2.65	2.72	0.033	<0.001	0.113	<0.001
C_8:0_	2.93	2.87	2.89	3.01	2.83	0.069	3.09 ^A^	2.84 ^B^	2.78 ^B^	0.036	0.409	<0.001	0.409
C_10:0_	9.54	9.45	9.52	9.9	9.52	0.188	10.09 ^A^	9.33 ^B^	9.35 ^B^	0.111	0.398	<0.001	0.368
C_11:0_	0.43	0.41	0.44	0.42	0.41	0.019	0.40 ^B^	0.42 ^B^	0.44 ^A^	0.009	0.638	<0.001	0.404
C_12:0_	5.34	5.53	5.51	5.78	5.64	0.167	5.87 ^A^	5.47 ^B^	5.33 ^B^	0.090	0.423	<0.001	0.347
C_13:0_	0.09	0.10	0.10	0.11	0.12	0.015	0.10	0.11	0.10	0.009	0.506	0.428	0.080
C_14:0_	12.51	12.64	12.54	12.71	12.68	0.259	11.99 ^C^	12.37 ^B^	13.49 ^A^	0.131	0.977	<0.001	0.263
C_14:1_	0.48 ^b^	0.57 ^a^	0.44 ^b^	0.36 ^c^	0.42 ^b^	0.021	0.51 ^A^	0.44 ^B^	0.41 ^B^	0.013	<0.001	<0.001	<0.001
C_15:0_	1.02	1.07	1.05	1.05	1.15	0.039	1.05 ^B^	1.04 ^B^	1.11 ^A^	0.020	0.238	<0.001	0.174
C_15:1_	0.29	0.31	0.30	0.30	0.31	0.013	0.30	0.30	0.30	0.007	0.686	0.514	0.168
C_16:0_	28.59	28.04	28.60	27.83	28.17	0.455	26.68 ^B^	27.02 ^B^	31.04 ^A^	0.248	0.691	<0.001	0.381
C_16:1_	1.53	1.51	1.67	1.56	1.63	0.053	1.43 ^C^	1.59 ^B^	1.72 ^A^	0.030	0.157	<0.001	0.232
C_17:0_	0.74	0.76	0.80	0.75	0.82	0.046	0.84 ^A^	0.86 ^A^	0.61 ^B^	0.024	0.707	<0.001	0.550
C_17:1_	0.28	0.28	0.30	0.28	0.31	0.017	0.32 ^A^	0.32 ^A^	0.23 ^B^	0.009	0.704	<0.001	0.531
C_18:0_	5.84	6.19	5.55	6.11	6.51	0.247	6.12 ^B^	6.36 ^A^	5.64 ^C^	0.129	0.066	<0.001	0.208
C_18:1_ Σ *trans* ^d^	0.46	0.42	0.42	0.44	0.44	0.019	0.43	0.45	0.42	0.011	0.511	0.121	0.023
C_18:1_ *t11*	1.55	1.32	1.25	1.28	1.18	0.092	1.41 ^A^	1.34 ^A^	1.20 ^B^	0.047	0.068	<0.001	0.859
C_18:1_ *c9*	16.87	17.06	17.78	17.46	17.92	0.475	18.23 ^A^	18.54 ^A^	15.24 ^B^	0.295	0.577	<0.001	0.294
C_18:2n6t_	0.32 ^a^	0.29 ^ab^	0.28 ^b^	0.27 ^b^	0.27 ^b^	0.012	0.30 ^A^	0.29 ^AB^	0.27 ^B^	0.007	0.021	<0.001	0.051
C_18:2n6c_	3.38	3.29	3.14	3.14	3.30	0.120	3.58 ^A^	3.42 ^B^	2.76 ^C^	0.058	0.558	<0.001	0.067
C_18:3n6_	0.03 ^a^	0.01 ^b^	0.01 ^bc^	0.03 ^a^	0.03 ^ac^	0.007	0.04 ^A^	0.02 ^A^	0.01 ^B^	0.004	0.010	<0.001	0.024
C_20:0_	0.11 ^a^	0.10 ^ab^	0.10 ^ab^	0.10 ^b^	0.09 ^c^	0.004	0.10 ^B^	0.09 ^C^	0.11 ^A^	0.003	0.002	<0.001	<0.001
C_18:3n3_	0.81 ^b^	0.82 ^ab^	0.78 ^b^	0.74 ^b^	0.90 ^a^	0.030	0.79 ^B^	0.84 ^A^	0.80 ^B^	0.015	0.007	<0.001	<0.001
*c9, t11* C_18:2_	0.94	0.80	0.84	0.79	0.76	0.055	0.83 ^B^	0.88 ^A^	0.76 ^C^	0.028	0.207	<0.001	0.170
C_20:3n3_	0.29 ^a^	0.28 ^a^	0.26 ^a^	0.22 ^b^	0.28 ^a^	0.011	0.27 ^A^	0.25 ^B^	0.28 ^A^	0.007	<0.001	0.006	<0.001
SCFA	18.41 ^a^	18.43 ^a^	18.55 ^a^	18.55 ^a^	16.95 ^b^	0.305	18.83 ^A^	18.00 ^B^	17.72 ^B^	0.182	0.001	<0.001	0.034
ΜCFA	47.55	47.37	47.80	47.49	47.76	0.672	45.69 ^B^	46.02 ^B^	51.07 ^A^	0.357	0.991	<0.001	0.131
LCFA	6.68	7.05	6.44	6.96	7.42	0.268	7.06 ^A^	7.31 ^A^	6.36 ^B^	0.140	0.099	<0.001	0.237
ΜUFA	21.44	21.47	21.75	21.67	22.20	0.500	22.63 ^A^	22.98 ^A^	19.51 ^B^	0.299	0.824	<0.001	0.247
PUFA	5.7	5.49	5.31	5.19	5.53	0.186	5.80 ^A^	5.70 ^A^	4.87 ^B^	0.090	0.241	<0.001	0.023
SFA	27.20	26.96	27.06	26.86	27.74	0.583	28.43 ^A^	28.68 ^A^	24.38 ^B^	0.329	0.842	<0.001	0.152
UFA	72.65	72.86	72.78	72.99	72.12	0.582	71.57 ^B^	71.32 ^B^	75.16 ^A^	0.328	0.849	<0.001	0.139
SFA/UFA	2.72	2.76	2.76	2.77	2.64	0.080	2.57 ^B^	2.51 ^B^	3.11 ^A^	0.043	0.768	<0.001	0.039
AI	3.15	3.20	3.22	3.22	3.11	0.115	2.90 ^B^	2.89 ^B^	3.74 ^A^	0.060	0.948	<0.001	0.051
Δ^−9^ desaturase index													
C_14:1_/C_14:0_	0.04 ^b^	0.05 ^a^	0.04 ^bc^	0.03 ^d^	0.03 ^c^	0.001	0.04 ^A^	0.04 ^B^	0.03 ^C^	0.001	<0.001	<0.001	<0.001
C_16:1_/C_16:0_	0.05	0.05	0.06	0.06	0.06	0.002	0.05 ^B^	0.06 ^A^	0.06 ^B^	0.001	0.160	<0.001	0.005
C_18:1_ c9/C_18:0_	2.92 ^ab^	2.78 ^b^	3.18 ^a^	2.89 ^b^	2.80 ^b^	0.094	3.02 ^A^	2.97 ^A^	2.75 ^B^	0.054	0.035	<0.001	0.752
*c9*, *t11*C_18:2/_*t11* C_18:1_	0.61 ^b^	0.62 ^ab^	0.69 ^a^	0.62 ^b^	0.67 ^ab^	0.020	0.60 ^B^	0.67 ^A^	0.65 ^A^	0.012	0.030	<0.001	0.663

Means with different superscript (a, b, c, d) in each row (between dietary treatments) and (A, B, C) (between sampling times) for each fatty acid differ significantly (*p* ≤ 0.05). ^a^ days; ^b^ SEM: Standard error of the mean; ^c^ Effect: The dietary treatment (T), time (S), and the interaction between dietary treatment × time (T × S) effects were analyzed by ANOVA using a general linear model (GLM) for repeated measures, and Post hoc analysis was performed when appropriate using Tukey multiple range test. ^d^ trans-11 C_18:1_ is not included in the Σ trans C_18:1_ content. C: basal diet (control); M: basal diet + 6 g/ewe RP methionine (MetaSmart™); L: basal diet + 5 g/ewe RP lysine (LysiGEM™); LML: basal diet + 6 g/ewe MetaSmart™ + 5 g/ewe LysiGEM™; and HML: basal diet + 12 g/ewe MetaSmart™ + 5 g/ewe LysiGEM™.

## Data Availability

Data are contained within the article and Supplementary Materials.

## Supplementary Materials

The following are available online at https://www.mdpi.com/article/10.3390/antiox10050654/s1, Figure S1. The mean individual protein and lactose content of ewes’ milk were fed with the five diets (C, M, L, LML, and HML) illustrated as connected superimposed symbols (interaction between the means of five dietary treatments and time response). Figure S2. The mean individual fatty acids (FA) (% of total FA) of ewes’ milk were fed with the five diets (C, M, L, LML, and HML) illustrated as connected superimposed symbols (interaction between the means of five dietary treatments and time response). Figure S3. The mean individual antioxidant enzyme activities and oxidative indices of ewes’ blood plasma were fed with the five diets (C, M, L, LML, and HML) illustrated as connected superimposed symbols (interaction between the means of five dietary treatments and time response). Figure S4. The mean individual antioxidant enzyme activities and oxidative indices of ewes’ milk were fed with the five diets (C, M, L, LML, and HML) illustrated as connected superimposed symbols (interaction between the means of five dietary treatments and time response). Table S1. Means of total antioxidant capacity, oxidative status biomarkers, and enzyme activities (Units/mL), in the blood plasma and milk of ewes fed the five diets (C, M, L, LML, and HML) within the three sampling times.

## References

[1] Schwab C.G., Broderick G.A. (2017). A 100-Year Review: Protein and amino acid nutrition in dairy cows. J. Dairy Sci..

[2] Liu Y.G., Peng H.H., Schwab C.G. (2013). Enhancing the productivity of dairy cows using amino acids. Anim. Prod. Sci..

[3] Tsiplakou E., Mavrommatis A., Kalogeropoulos T., Chatzikonstantinou M., Koutsouli P., Sotirakoglou K., Labrou N., Zervas G. (2017). The effect of dietary supplementation with rumen-protected methionine alone or in combination with rumen-protected choline and betaine on sheep milk and antioxidant capacity. J. Anim. Physiol. Anim. Nutr..

[4] Arriola Apelo S.I., Bell A.L., Estes K., Ropelewski J., de Veth M.J., Hanigan M.D. (2014). Effects of reduced dietary protein and supplemental rumen-protected essential amino acids on the nitrogen efficiency of dairy cows. J. Dairy Sci..

[5] Bröer S., Bröer A. (2017). Amino acid homeostasis and signalling in mammalian cells and organisms. Biochem. J..

[6] Yin J., Li T., Yin Y. (2016). Methionine and Antioxidant Potential. J. Antioxid. Act..

[7] Bequette B.J., Backwell F.R., Kyle C.E., Calder A.G., Buchan V., Crompton L.A., France J., MaCrae J.C. (1999). Vascular sources of phenylalanine, tyrosine, lysine, and methionine for casein synthesis in lactating goats. J. Dairy Sci..

[8] Glascock R.F., Welch V.A. (1974). Contribution of the fatty acids of three low density serum lipoproteins to bovine milk fat. J. Dairy Sci..

[9] Park J.K., Yeo J.M., Bae G.S., Kim E.J., Kim C.H. (2020). Effects of supplementing limiting amino acids on milk production in dairy cows consuming a corn grain and soybean meal-based diet. J. Anim. Sci. Technol..

[10] Chow J.M., DePeters E.J., Baldwin R.L. (1990). Two Rumen-Protected Amino Acids in Dairy Cows’ Feed Change the Protein Content Of Milk. Calif. Agric..

[11] Li P., Yin Y.L., Li D., Kim S.W., Wu G. (2007). Amino acids and immune function. Br. J. Nutr..

[12] Sun F., Cao Y., Cai C., Li S., Yu C., Yao J. (2016). Regulation of Nutritional Metabolism in Transition Dairy Cows: Energy Homeostasis and Health in Response to Post-Ruminal Choline and Methionine. PLoS ONE.

[13] Lopes M.G., Dominguez J.H.E., Corrêa M.N., Schmitt E., Fischer G. (2019). Rumen-protected methionine in cattle: Influences on reproduction, immune response, and productive performance. Arq. Inst. Biol..

[14] Awawdeh M.S. (2016). Rumen-protected methionine and lysine: Effects on milk production and plasma amino acids of dairy cows with reference to metabolisable protein status. J. Dairy Res..

[15] Vailati-Riboni M., Zhou Z., Jacometo C.B., Minuti A., Trevisi E., Luchini D.N., Loor J.J. (2017). Supplementation with rumen-protected methionine or choline during the transition period influences whole-blood immune response in periparturient dairy cows. J. Dairy Sci..

[16] Coleman D.N., Lopreiato V., Alharthi A., Loor J.J. (2020). Amino acids and the regulation of oxidative stress and immune function in dairy cattle. J. Anim. Sci..

[17] Toledo M.Z., Baez G.M., Garcia-Guerra A., Lobos N.E., Guenther J.N., Trevisol E., Luchini D., Shaver R.D., Wiltbank M.C. (2017). Effect of feeding rumen-protected methionine on productive and reproductive performance of dairy cows. PLoS ONE.

[18] Tsiplakou E., Mavrommatis A., Skliros D., Righi F., Flemetakis E. (2020). The impact of rumen-protected amino acids on the expression of key- genes involved in the innate immunity of dairy sheep. PLoS ONE.

[19] Tsiplakou E., Mavrommatis A., Skliros D., Sotirakoglou K., Flemetakis E., Zervas G. (2018). The effects of dietary supplementation with rumen-protected amino acids on the expression of several genes involved in the immune system of dairy sheep. J. Anim. Physiol. Anim. Nutr..

[20] Ali C.S., Islam-ud-Din, Sharif M., Nisa M., Javaid A., Hashmi N., Sarwar M. (2009). Supplementation of ruminally protected proteins and amino acids: Feed consumption, digestion and performance of cattle and sheep. Int. J. Agric. Biol..

[21] Cannas A., Tedeschi L.O., Fox D.G., Pell A.N., Van Soest P.J. (2004). A mechanistic model for predicting the nutrient requirements and feed biological values for sheep1. J. Anim. Sci..

[22] (1993). IDF: Bulletin of the IDF No. 285/1993—Reference Materials and Interlaboratory Collaborative Studies (Third Series), by Various Groups of Experts (See Also Bulletins Nos 207/1986, 235/1988).

[23] The Royal Society of Chemistry British Standards Institution. https://pubs.rsc.org/en/Content/ArticleLanding/1952/AN/an952770546a#!divAbstract.

[24] ISO (International Organization for Standardization) (2004). Milk—Determination of Casein-Nitrogen Content—Part 1: Indirect Method (ISO 17997-1/IDF 29).

[25] Bocquier F., Barillet F., Guillouet P., Jacquin M. (1993). Pre’vision de l’energie du lair de brebis a` partir de diffe’rents re’sultats d’ analyses: Proposition de lait standard pour les brebis laitie`res. Ann. Zootech..

[26] Mavrommatis A., Tsiplakou E. (2020). The impact of the dietary supplementation level with Schizochytrium sp. on milk chemical composition and fatty acid profile, of both blood plasma and milk of goats. Small Rumin. Res..

[27] Tsiplakou E., Mitsiopoulou C., Mavrommatis A., Karaiskou C., Chronopoulou E.G., Mavridis G., Sotirakoglou K., Labrou N.E., Zervas G. (2018). Effect of under- and overfeeding on sheep and goat milk and plasma enzymes activities related to oxidation. J. Anim. Physiol. Anim. Nutr..

[28] Paglia D.E., Valentine W.N. (1967). Studies on the quantitative and qualitative characterization of erythrocyte glutathione peroxidase. J. Lab. Clin. Med..

[29] Nielsen F., Mikkelsen B.B., Nielsen J.B., Andersen H.R., Grand-Jean P. (1997). Plasma malondialdehyde as bio-marker for oxidative stress: Reference interval and effects of life-style factors. Clin. Chem..

[30] Mavrommatis A., Mitsiopoulou C., Christodoulou C., Karabinas D., Nenov V., Zervas G., Tsiplakou E. (2020). Dietary Supplementation of a Live Yeast Product on Dairy Sheep Milk Performance, Oxidative and Immune Status in Peripartum Period. J. Fungi.

[31] Patsoukis N., Zervoudakis G., Panagopoulos N.T., Georgiou C.D., Angelatou F., Matsokis N.A. (2004). Thiol redox state (TRS) and oxidative stress in the mouse hippocampus after pentylenetetrazolinduced epileptic seizure. Neurosci. Lett..

[32] Pellegrini N., Serafini M., Colombi B., Del Rio D., Salvatore S., Bianchi M., Brighenti F. (2003). Total antioxidant capacity of plant foods, beverages and oils consumed in Italy assessed by three different in vitro assays. J. Nutr..

[33] Li P., Huo L., Su W., Lu R., Deng C., Li L., He C. (2011). Free radical scavenging capacity, antioxidant activity and phenolic content of Pouzolzia zeylanica. J. Serbian Chem. Soc..

[34] Benzie I.F., Strain J.J. (1996). The ferric reducing ability of plasma (FRAP) as a measure of “antioxidant power”: The FRAP assay. Anal. Biochem..

[35] Baldwin J.A., Horton G.M.J., Wohlt J.E., Palatini D.D., Emanuele S.M. (1993). Rumen-protected methionine for lactation, wool and growth in sheep. Small Rumin. Res..

[36] Lara A., Mendoza G.D., Landois L., Barcena R., Sánchez-Torres M.T., Rojo R., Vega S. (2006). Milk production in Holstein cows supplemented with different levels of ruminally protected methionine. Livest. Sci..

[37] Třináctý J., Křížová L., Richter M., Černý V., Říha J. (2009). Effect of rumen-protected methionine, lysine or both on milk production and plasma amino acids of high-yielding dairy cows. Czech J. Anim. Sci..

[38] Chen Z.H., Broderick G.A., Luchini N.D., Sloan B.K., Devillard E. (2011). Effect of feeding different sources of rumen-protected methionine on milk production and N-utilization in lactating dairy cows1. J. Dairy Sci..

[39] Rulquin H., Vérité R., Garnsworthy P.C., Cole D.J.A. (1996). Amino acid nutrition of dairy cows: Productive effects and animal requirements. Recent Developments in Animal Nutrition.

[40] Oldham J.D., Phillips C.J.C. (1996). Protein requirement system for ruminants. Progress in Dairy Science.

[41] St-Gelais D., Haché S. (2005). Effect of β-casein concentration in cheese milk on rennet coagulation properties, cheese composition and cheese ripening. Food Res. Int..

[42] Goulas C., Zervas G., Papadopoulos G. (2003). Effect of dietary animal fat and methionine on dairy ewes milk yield and milk composition. Anim. Feed Sci. Technol..

[43] Flores A., Mendoza G., Pinos-Rodrıguez J.M., Plata F., Vega S., Barcena R. (2009). Effects of rumen-protected methionine on milk production of dairy goats. Ital. J. Anim. Sci..

[44] Poljicak-Milas N., Marenjak T.S. (2007). Dietary supplement of the rumen protected methionine and milk yield in dairy Goats. Arch. Tierz..

[45] Al-Qaisi M.A., Titi H.H. (2014). Effect of rumen-protected methionine on production and composition of early lactating Shami goats milk and growth performance of their kids. Arch. Tierz..

[46] Kudrna V., Illek J., Marounek M., Nguyen Ngoc A. (2009). Feeding ruminally protected methionine to pre- and postpartum dairy cows: Effect on milk performance, milk composition and blood parameters. Czech J. Anim. Sci..

[47] Yang W.R., Sun H., Wang Q.Y., Liu F.X., Yang Z.B. (2010). Effects of rumen protected methionine on dairy performance and amino acid metabolism in lactating cows. Am. J. Anim. Vet. Sci..

[48] Soltan M.A., Mujalli A.M., Mandour M.A., El-Shinaway Abeer M. (2012). Effect of dietary rumen protected methionine and/or choline supplementation on rumen fermentation characteristics and productive performance of early lactating cows. Pak. J. Nutr..

[49] Mepham T.B. (1982). Amino acid utilization by lactating mammary gland. J. Dairy Sci..

[50] Bequette B.J., Backwell F.R., Crompton L.A. (1998). Current concepts of amino acid and protein metabolism in the mammary gland of the lactating ruminant. J. Dairy Sci..

[51] Bionaz M., Periasamy K., Rodriguez-Zas S.L., Everts R.E., Lewin H.A., Hurley W.L., Loor J.J. (2012). Old and new stories: Revelations from functional analysis of the bovine mammary transcriptome during the lactation cycle. PLoS ONE.

[52] Garnsworthy P.C., Masson L.L., Lock A.L., Mottram T.T. (2006). Variation of milk citrate with stage of lactation and de novo fatty acid synthesis in dairy cow. J. Dairy Sci..

[53] Faulkner A., Peaker M. (1982). Reviews of the progress of dairy science: Secretion of citrate into milk. J. Dairy Res..

[54] Sevi A., Rotunno T., Di Caterina R., Muscio A. (1998). Rumen-protected methionine or lysine supplementation of Comisana ewes’ diets: Effects on milk fatty acid composition. J. Dairy Res..

[55] Giallongo F., Harper M.T., Oh J., Lopes J.C., Lapierre H., Patton R.A., Parys C., Shinzato I., Hristov A.N. (2016). Effects of rumen-protected methionine, lysine, and histidine on lactation performance of dairy cows. J Dairy Sci..

[56] Feng L., Xiao X.X., LIU Y., Jiang J., Hu K., Jiang W.D., Li S.H., Zhou X.Q. (2011). Methionine hydroxy analogue prevents oxidative damage and improves antioxidant status of intestine and hepatopancreas for juvenile Jian carp (*Cyprinus carpio var*. Jian). Aquac. Nutr..

[57] Osorio J.S., Trevisi E., Ji P., Drackley J.K., Luchini D., Bertoni G., Loor J.J. (2014). Biomarkers of inflammation, metabolism, and oxidative stress in blood, liver, and milk reveal a better immunometabolic status in peripartal cows supplemented with Smartamine M or MetaSmart. J. Dairy Sci..

[58] Wang S.T., Chen H.W., Sheen L.Y., Lii C.K. (1997). Methionine and Cysteine Affect Glutathione Level, Glutathione-Related Enzyme Activities and the Expression of Glutathione S-Transferase Isozymes in Rat Hepatocytes. J. Nutr..

[59] D’Angelo A., Selhub J. (1997). Homocysteine and thrombotic disease. Blood.

[60] Pizzorno J. (2014). Glutathione!. Integr. Med. (Encinitas).

[61] Gould R.L., Pazdro R. (2019). Impact of Supplementary Amino Acids, Micronutrients, and Overall Diet on Glutathione Homeostasis. Nutrients.

[62] Jankowski J., Ognik K., Kubińska M., Czech A., Juśkiewicz J., Zduńczyk Z. (2017). The effect of DL-, L-isomers and DL-hydroxy analog administered at 2 levels as dietary sources of methionine on the metabolic and antioxidant parameters and growth performance of turkeys. Poult. Sci..

[63] Kapusta A., Kuczyńska B., Puppel K. (2018). Relationship between the degree of antioxidant protection and the level of malondialdehyde in high-performance Polish Holstein-Friesian cows in peak of lactation. PLoS ONE.

[64] Esterbauer H., Schaur R.J., Zollner H. (1991). Chemistry and biochemistry of 4-hydroxynonenal, malonaldehyde and related aldehydes. Free Radic. Biol. Med..

[65] Jové M., Mota-Martorell N., Pradas I., Martín-Gari M., Ayala V., Pamplona R. (2020). The Advanced Lipoxidation End-Product Malondialdehyde-Lysine in Aging and Longevity. Antioxidants.

[66] Chio K.S., Tappel A.L. (1969). Synthesis and characterization of the fluorescent products derived from malonaldehyde and amino acids. Biochemistry.

[67] Sayre L.M., Moreira P.I., Smith M.A., Perry G. (2005). Metal ions and oxidative protein modification in neurological disease. Ann. Ist. Super. Sanita.

[68] Yuan S.B., Chen D.W., Zhang K.Y., Yu B. (2007). Effects of oxidative stress on growth performance, nutrient digestibilities and activities of antioxidative enzymes of weanling pigs. Asian Australas. J. Anim. Sci..

