# Supplementary material for: Effects of Supplementing Rumen-Protected Methionine and Lysine on Milk Performance and Oxidative Status of Dairy Ewes
Source: Antioxidants (Basel). 2021 Apr 23;10(5):654. doi: 10.3390/antiox10050654 (PMC8147003; doi:10.3390/antiox10050654)
Supplement: Supplementary file 1 [file antioxidants-10-00654-s001.zip › antioxidants-1158781-supplementary.pdf]

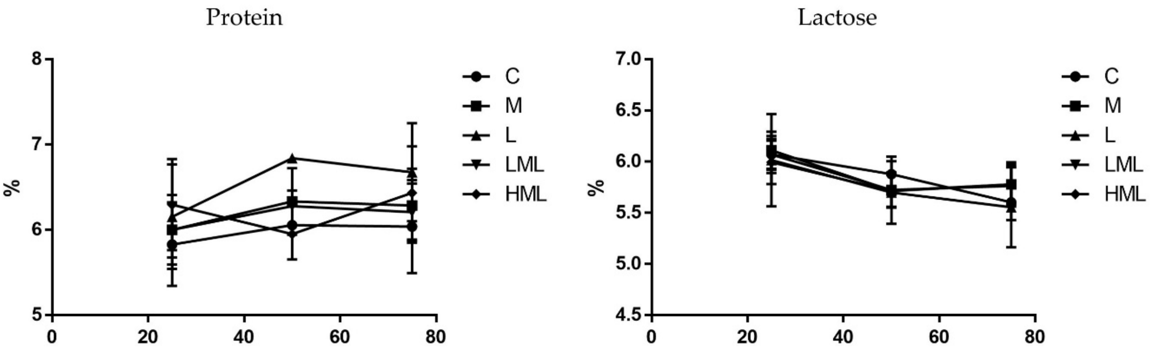

Figure S1. The mean individual protein and lactose content of ewes' milk were fed with the five diets (C, M, L, LML, and HML) illustrated as connected superimposed symbols (interaction between the means of five dietary treatments and time response).

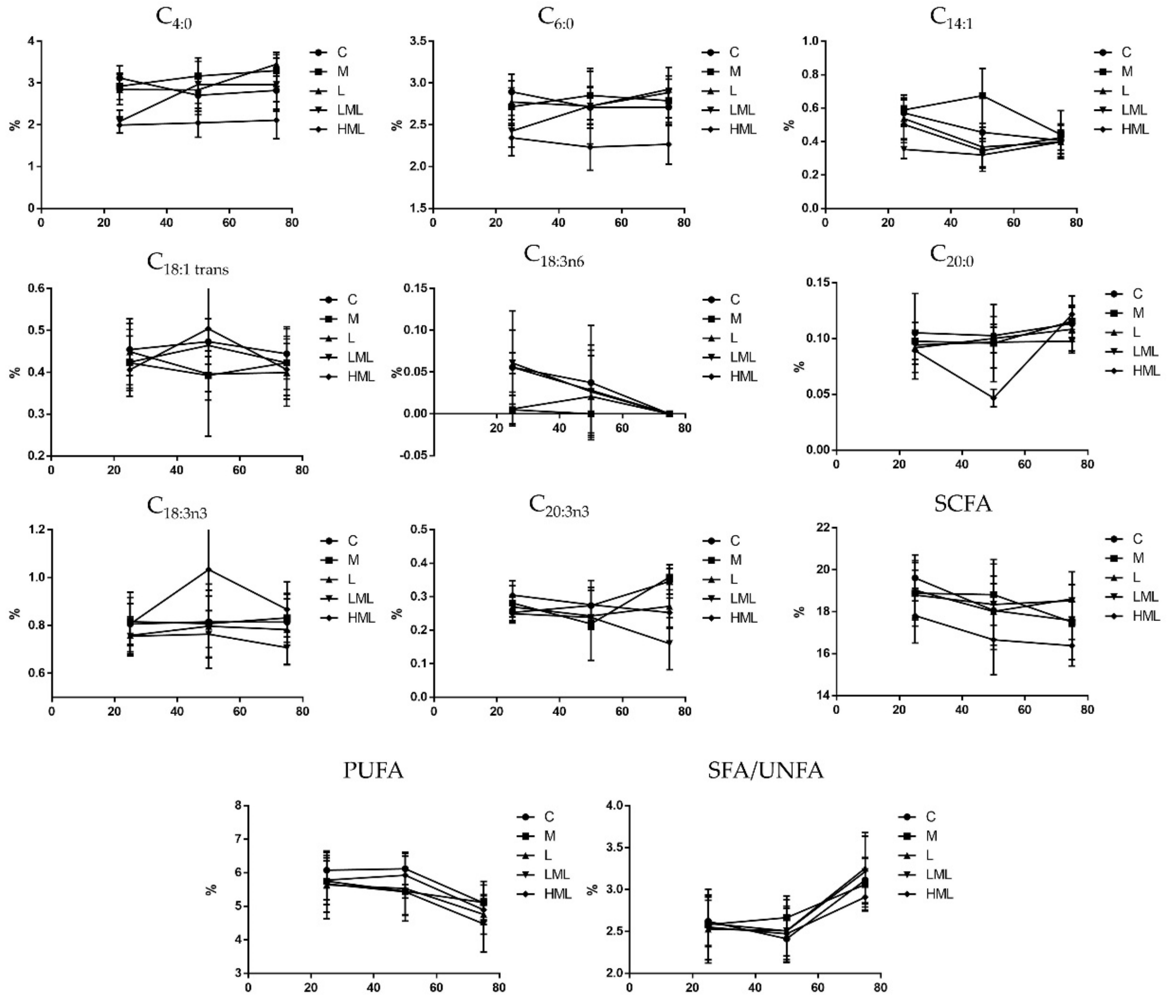

Figure S2. The mean individual fatty acids (FA) (% of total FA) of ewes' milk were fed with the five diets (C, M, L, LML, and HML) illustrated as connected superimposed symbols (interaction between the means of five dietary treatments and time response).

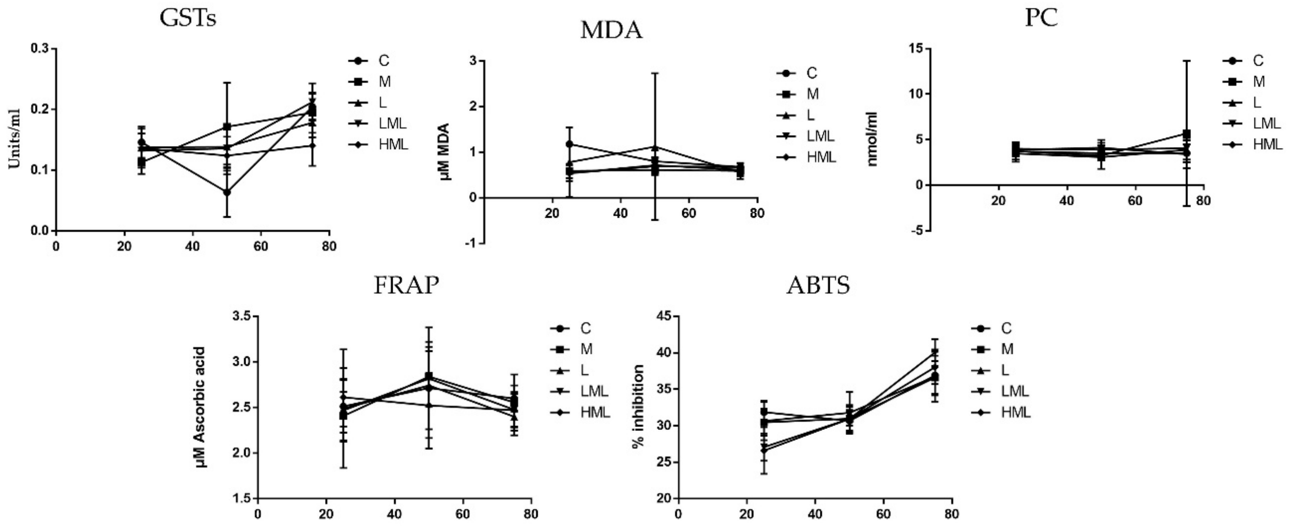

Figure S3. The mean individual antioxidant enzyme activities and oxidative indices of ewes' blood plasma were fed with the five diets (C, M, L, LML, and HML) illustrated as connected superimposed symbols (interaction between the means of five dietary treatments and time response).

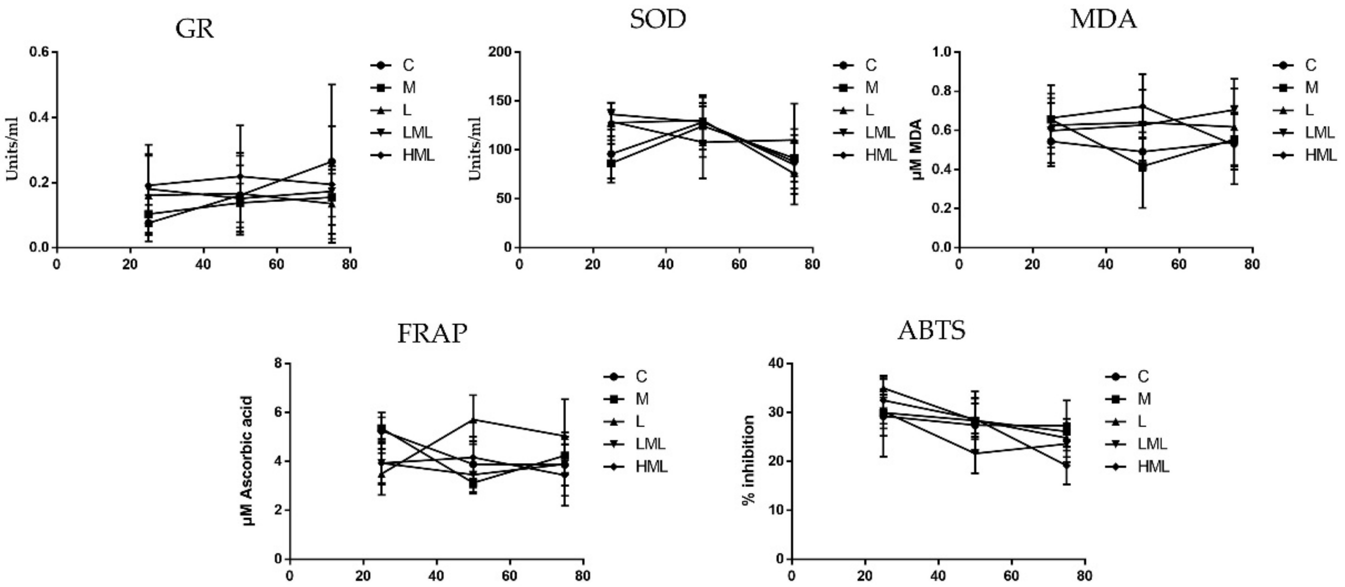

Figure S4. The mean individual antioxidant enzyme activities and oxidative indices of ewes' milk were fed with the five diets (C, M, L, LML, and HML) illustrated as connected superimposed symbols (interaction between the means of five dietary treatments and time response).

Table S1. Means of total antioxidant capacity, oxidative status biomarkers, and enzyme activities (Units/mL), in the blood plasma and milk of ewes fed the five diets (C, M, L, LML, and HML) within the three-sampling time.

	Diets (D)						Sampling time (T)				Effect‡		
	**C**	**M**	**L**	**LML**	**HML**	**SEM[1]**	**25**	**50**	**75**	**SEM[1]**	**D**	**T**	**D x T**
							Blood						
[2]CAT	4.12	3.70	4.02	4.62	3.64	0.374	5.33^{a}	3.52^{b}	3.22^{b}	0.237	NS	***	NS
[3]GSH-Px	0.08	0.09	0.09	0.08	0.08	0.006	0.08	0.09	0.09	0.003	NS	NS	NS
[4]GR	0.04	0.05	0.04	0.05	0.04	0.012	0.05^{a}	0.03^{b}	0.04^{ab}	0.008	NS	***	NS
[5]SOD	9.24	8.92	8.92	8.85	8.91	0.320	8.42^{a}	8.83^{ab}	9.66^{b}	0.189	NS	***	NS
[6]GST	0.14^{a}	0.16^{b}	0.15^{ab}	0.16^{b}	0.13^{a}	0.006	0.13^{a}	0.13^{a}	0.19^{b}	0.004	**	***	***
[7]MDA	0.89^{a}	0.60^{b}	0.82^{ab}	0.62^{b}	0.64^{b}	0.108	0.73	0.79	0.62	0.047	*	NS	***
[8]PC	3.62	3.37	3.53	3.99	3.81	0.408	3.76	3.64	4.12	0.231	NS	NS	NS
[9]FRAP	2.55	2.60	2.56	2.59	2.54	0.079	2.49^{a}	2.71^{b}	2.50^{a}	0.045	NS	***	*
[10]ABTS	33.20	32.70	33.17	32.69	31.77	0.527	29.39^{a}	31.06^{b}	37.67^{c}	0.310	NS	***	***
							Milk						
[4]GR	0.17	0.13	0.15	0.17	0.20	0.026	0.14	0.17	0.19	0.015	NS	NS	*
[5]SOD	104.18^{t}	100.89	115.72^{t}	117.03^{t}	111.63	5.235	114.76^{a}	123.33^{ab}	89.87^{b}	3.787	T	***	***
[11]LPO	0.55	0.52	0.52	0.53	0.49	0.070	0.58^{a}	0.55^{ab}	0.45^{b}	0.039	NS	***	NS
[7]MDA	0.53^{a}	0.55^{ab}	0.63^{b}	0.64^{b}	0.64^{b}	0.033	0.62	0.58	0.59	0.018	*	NS	**
[8]PC	1.49^{ac}	1.72^{c}	2.04^{b}	1.60^{cb}	1.81^{b}	0.105	1.81^{b}	1.93^{b}	1.45^{a}	0.089	**	**	NS
[9]FRAP	4.33^{a}	4.25^{a}	4.82^{b}	3.64^{c}	3.73^{c}	0.201	4.39^{a}	4.03^{b}	4.05^{b}	0.113	**	*	***
[10]ABTS	28.88^{a}	27.34^{a}	29.53^{a}	25.14^{b}	26.72^{ab}	0.854	31.45^{a}	26.74^{b}	24.37^{c}	0.649	**	***	***

Means with different superscript (a, b, c) between dietary treatments and (a, b, c) between sampling time differ significantly ($p \leq 0.05$).

‡ Effect: The dietary treatment (D), time (T), and the interaction between dietary treatment x time (DxT) effects were analyzed by ANOVA using a general linear model (GLM) for repeated measures and Post hoc analysis was performed when appropriate using Turkey's multiple range test.

*p < 0,05, **p < 0,01, ***p < 0,001, NS= no significant, t = tendency, P-value < 0.10.

[1]SEM: Standard error of means

[2]CAT: Catalase

[3]GSH-Px: Glutathione peroxidase

[4]GR: Glutathione reductase
[5]SOD: Superoxide dismutase
[6]GST: Glutathione transferase
[7]MDA: Malondialdehyde (μM MDA)
[8]PC: Protein Carbonyls (nmol/ml)
[9]FRAP: Ferric Reducing Ability of Plasma (μM ascorbic acid)
[10]ABTS: 2,2'-azino-di (3-ethylbenzthiazoline-6-sulfonic acid (inhibition %)
[11]LPO: Lactoperoxidase
